# Notes of a Case of Sclerotitis Apparently of Dental Origin

**Published:** 1889-11

**Authors:** John Hern

**Affiliations:** Ophthalmic Surgeon Darlington Hospital.


ARTICLE IV.

### NOTES OF A CASE OF SCLEROTITIS APPARENTLY OF DENTAL ORIGIN.

BY JOHN HERN, M. D. EDIN., M. R. C. S. ENG.,
Ophthalmic Surgeon Darlington Hospital.

[Read in the Section of Ophthalmology at the Annual Meeting of the British Medical Association, held in Leeds, August, 1889.]

It is with considerable reluctance that I decided to bring a single case before the members of this section, but ultimately resolved to do so, thinking it probable that other cases of the kind have been met with, though, so far as I can certain, not published. My attention was drawn to the dental organs as a reflex cause of ophthalmic affections by an excellent paper read before the Odontological Society (and published in its reports), by Mr. Power; but it is evident from it that sclerotitis has not been noticed by him as one of the affections of probable dental origin.

Mrs. Y., aged 38, a healthy, well-developed woman, the mother of three healthy children; no history or other signs or symptoms of syphilis or rheumatism, or any other morbid constitutional tendency. The husband and relations are also well known to me, and have no particular constitutional tendency to disease.

On March 27th, 1888, Mrs. Y., consulted me for a severe pain referred to the point of emergence of the left infra-orbital branch of the superior maxillary division of the fifth, and shooting up over the nasal bone; this pain came on in paroxysms several times during the day, and, as the patient described it, "inflamed her eye." On examining the left eye, tension seemed slightly above the normal, but, on comparing it with the right, they were found to be as nearly as possible the same; the eye was not tender to the touch, but at the upper margin of the cornea, about two lines external to the vertical meridian, a distinct patch of sclerotitis was observed, the contiguous corneal substance being quite hazy in its superficial layers. The patch at this time was about the size of the flat surface of a split pea. Although six-grain doses of quinine several times a day relieved the pain, the patch gradually spread outwards and downwards along the corneal margin, involving its superficial layers almost to the pupillary margin. I noticed the patch spread and enlarged most on those days when the patient complained of most pain referred to the infraorbital region.

In August, 1888, my notes of the case read as follows:—"Sclerotitic patch has spread from the vertical meridian above to four lines internal to vertical meridian below, and has also extended two lines nearer the posterior pole than when first seen five months ago. Tension plus ½ to 1 L. Pain now referred to all three divisions of the fifth (supra-orbital, infra-orbital and the mental branch of the inferior dental); also pain in the upper and lower teeth of left side, especially the former. Patient complains that on taking anything, either hot or cold, into the mouth, the teeth ache (especially the left uppers), and that pains shoot up into the left eye."

The condition of the eye became gradually worse until late in the following month (September). There being an increase in the pain referred to the teeth, I asked my brother, William Hern, who was spending his holiday with me, to see the case, which he accordingly did, and discovered an exposed nerve in the second left upper bicuspid, and at once advised its removal, considering it the probable cause of the pain. The vision of the left eye was carefully tested late in September, and found to be 20-30 L. Tension + 1 L.

On October 5th, the tooth was extracted, and sent direct to my brother for a report on its *post mortem* condition, which I will give in his own words:—"Upper second bicuspid of left side affected with a very large and deep carious cavity on its anterior surface, involving the pulp chamber, and a smaller and shallower one on its posterior surface. On splitting, the pulp was found to have been alive, its free surface ulcerating and covered with thin greenish pus, its deeper parts intensely red and injected; the pulp tissue altogether being in a state of great irritation, and a potential cause of much pain, both local and reflex." He also says:—"I have seen several cases amongst my patients at the Dental and Middlesex Hospitals of undoubted reflex ophthalmic disturbance due to dental causes, in which the lesion was less clearly marked than in this case. In a few cases which have been sent me from Moorfields or from the out patient ophthalmic department of my own hospital, an exposed pulp in some lurking position has most commonly been found to be the irritating cause."

As to the progress of the case after the extraction of the tooth, the pain disappeared immediately and did not recur. For a fortnight the condition of the eye appeared to remain absolutely stationary, aad then gradually the lesion gave in, and disappeared in from six to eight weeks, leaving a slight haziness of the cornea, with the usual thinning of the sclerotic, giving the blue coloration of the choroidal pigment over the seat of the sclerotitic patch. Tension in L. became equal to that of the R., and sight returned to the extent of 28-30.

Having given a fair trial to all the recognised methods of treatment before the extraction of the tooth, I at that time ceased to administer all internal remedies, and simply applied a solution of boracic acid locally. Although the *post quod er go propter quod* is not a safe method of reasoning, yet the gradual spreading of the patch up to the time of the extraction of the tooth, the sudden check produced thereby, the gradual diminution, dating from a fortnight after the extraction, and, finally, the gradual return of the eye to its normal condition, point, I think, to the probability that in this case, at any rate, we have an example of an irritative lesion reflexly produced, in which the afferent nerve is the superior maxillary, or second division of the fifth nerve, and the efferent nerve the first division of the fifth and probably its nasal branch.—*British Medical Journal.*

